# Supplementary material for: Estimating the false discovery risk of (randomized) clinical trials in medical journals based on published p-values
Source: PLoS One. 2023 Aug 30;18(8):e0290084. doi: 10.1371/journal.pone.0290084 (PMC10468063; doi:10.1371/journal.pone.0290084)
Supplement: Attachment — Submitted filename: Response..docx [file pone.0290084.s001.docx]

Dear Dr. Bobrovitz,

We thank the reviewers for their detailed and thoughtful reviews. We appreciate the time invested to give feedback which helped in improving our manuscript to this revised version.

Below we have copied each point raised by our reviewers (in italics) with a response explaining how we changed our manuscript accordingly (in bold). All changes to the manuscript are highlighted in blue in the revised version.

**Reviewer #1:**

Minor points:

*it is a personal choice, but to me the starting reference to vaccines seems completely unnecessary and rather out of place. If the authors want to make the starting point that science works, they could refer to excellent academic articles like* [*https://doi.org/10.1073/pnas.1711786114*](https://doi.org/10.1073/pnas.1711786114)*.*

**We decided to remove this unnecessary part of our Introduction.**

*lines 40-42 it is "well known that" with no reference sounds very unconvincing to me. I would refer to the gigantic systematic reviews by Song et al (and perhaps there is something more recent but I doubt it will be that extensive). However, note that, according to Song et al, the evidence of the kind of problem the authors give for granted is not as firmly established as the authors state. In particular the author confound the issue of the file drawer problem (null results don't get published) with a causal assumption about what generates it (journals reject them), which is not well supported. I would change the sentence to something like "journals may suffer from a publication bias" or "from a selective reporting problem" and cite one or few reviews.*

**We no longer make this claim and simply discuss the problem of selection bias without making claims about its prevalence or magnitude.**

*Fairly important concerns:*

*I think that a deeper hidden assumption in this whole paragraph (and the whole analysis, really) is this dichotomy between "true" and "false" results, which is rather unrealistic. Perhaps I am missing something, but what does it mean to have "100% power"? Power is relative to a magnitude of effect size, and there is always a threshold that needs to be set to determine it. Effects smaller than whatever threshold is set are not "false" and yet will be less detectable. I would encourage the authors to discuss this assumption more, and refer to a recent analysis that - luckily for them - suggests that even relaxing this assumption the picture doesn't change substantially in terms of false discovery rates etc. see https://osf.io/preprints/metaarxiv/jk7sa/*

**We agree that the dichotomy between true and false findings is a problem for models that assume two population of studies as Jager and Leek’s model did. However, our model does not make this assumption and we solve this problem by estimating the maximum false positive rate which can be estimated using Soric’s approach. The assumption of 100% power is also idealistic and only used to estimate the maximum false positive rate. As actual mean power is likely to be lower, it is clear that this estimate is higher than the true false positive rate. 100% power could be achieved by conducting studies in which sampling error is always much lower than effect sizes producing high test statistics, z = ES / SE = 5 (5 sigma). While power is not strictly 100%, these studies are unlikely to produce type-2 errors (false negative results).**

*Lines 185 and following: first, the authors need to explain better the link between z-curve and replicability, and particualrly explain what they define as replicability (which is presumably linked to the chances of a replication falling within the confidence interval of the original, or other arbitrary criterion which is based on the same logic of P-values, and is not necessarily the correct criterion (see for example the writings of Gelman on how it wrong to think of replication in binary terms). But more importantly, the authors fail to mention the many replicability studies that, contrary to what they refer, do NOT show unambigiuously low replicability. This was discussed somehow in their reference n. 2 and also in Chapter 4 of this book: https://global.oup.com/academic/product/research-integrity-9780190938550?cc=gb&lang=en&#, but is also discussed in many of those same studies (e.g. experimental economics, experimental philosophy, cognitive science all concluded they do not have much of a problem, and their replicability rates only modestly higher than those in the other studies.*

*Again, the claim that reproducibility is too low depends on what expectation one should have, which is a matter of defining what standards and thresholds and assumptions one makes about changes in the population and methods etc. The authors mention some of this, but in my opinion they should either develop the argument in full as it deserves, or should remove this part, which once more seems to oversell the implications of the results.*

**Replicability is a secondary concern in the context of this paper. We provide a clearer definition of replicability as it relates to z-curve. This definition focusses on rejecting the null-hypothesis again using the same alpha criterion and the same sample size.**

***“Z-curve also provides information about the expected replication rate of significant***

***results in medical abstracts, defined as the probability of obtaining a statistically***

***significant result in the replication study (see e.g., [28–30] for alternatives).”***

**We discuss the implications of our results, but they remain secondary to our main goal of testing the prediction that most published results in medical journals are false.**

*More important concerns:*

*I feel that the article needs to provide in greater detail, to the general reader, what the methods used imply. The Z curve method deserves its own introduction, and so does the rest of the analysis. I happen to have known the Jager and Leek paper and therefore could grasp at least the generalities of the simulation methods used, but I wonder what the average reader outside Metascience can understand of all this.*

*There needs to be a formal Methods section that explains the modelling in detail.*

**We restructured the paragraph and provided a formal Methods section containing descriptions of false discovery risk and z-curve. We now provide more detailed description of the methods and also summarize the extensions we developed for this manuscript.**

*The authors need to be much more cautious about their results and interpretation. Apart from the hidden assumptions mentioned above and other modelling choices that may become more apparent once the methods are laid out in full, I can see at least two major critiques of their work, only one of which is discussed, and insufficiently in my opinion. The implications of these critiques are opposite to each other.*

*On the one hand, abstracts in top journals are likely not representative of the overall population of studies, not just because the methodological standards are likely higher, but also because the types of studies published in these journals are often meeting different assumptions from those made By Ioannidis 2005. I am thinking that many studies will be clinical trials, which are usually carried out when the level of uncertainty is at 50% (i.e. relative high priors). And many other studies in these journals are completing a research program, conclusively proving something that was believed for a long time and had mounting evidence (again, high priors). The priors may be much lower for hypotheses tested in lower-ranking journals. This would entail that their numbers may be grossly under-estimating the "bulk" of the problem.*

**We address this valid point by limiting our discussion to clinical trials. Ioannidis (2005) distinguished three scenarios of clinical trials. One assumed a low FDR (15%) that actually matches our risk estimate. The other scenarios assumed fewer true hypotheses and lower power (and either small or large amount of bias that only affects the observed discovery rate but not our estimates of the discovery rate). Our results would suggest that most of the clinical trials reported in the journals that we examined were high quality clinical trials. This is itself an important finding, but it is important to avoid generalizing it to all areas of science or medicine. Our article shows how this question can be answered empirically by conducting similar studies for other research areas. Unfortunately, Ioannidis’s article has been overgeneralized to all areas without noticing the differences in his scenarios. Our results show how important it is to conduct separate analyses for different fields of study.**

*On the other hand, one could also argue that abstract tend to over-represent small P-values relative to the fulltext they are summarizing, and therefore even these estimates are likely an over-estimate of the true rate of false positives.*

*Either way, it seems improper for the authors to draw general conclusions about "medical research" or even "top medical journals" the way they do repeatedly in the text.*

*The discussion opens with a long reflection on science and trust, which I feel slows down the pace of the article and would be better placed at the end of the discussion or in the introduction, if not removed entirely.*

**We acknowledge this limitation in the discussion section and point out that hand-coding or results in a field with a lot of researcher bias (social psychology) also does not support the conclusion that most results are false. Similar research in medical journals could help to address the concern about “abstract bias.”**

*I was a bit baffled by the conclusions of the authors who, after having announced in the abstract and suggested in the results that the false discovery rate is much much lower than what many fear, then adopt a dramatic and negative tone at the end, mainly enphasizing the dire implications of their findings. What is the problem with concluding that things may not be so bad after all? Wasn't that the point? And, more importantly, due to limitations that are currently only modestly discussed, the authors should be much more cautious about any conclusion they draw, one way or the other.*

**We revised the introduction to focus on the “good news” first. Yet, our evidence of selection bias provides some clear empirical evidence that results of published articles are selected (without making claims about the process) to be significant which means meta-analytic effect size estimates are inflated. We recommend new meta-analytic methods to correct for this bias and hope that journal editors will try to reduce this selection bias. Of course, inflation of effect sizes is not as problematic as a field that publishes mostly false results, but we do believe that the evidence of selection bias deserves to be mentioned.**

*In conclusion, I think that this work is certainly interesting and worthy of publication, but in my opinion it needs to be expanded and reshaped, by lowering the tones, avoiding the rhetoric, and instead focusing more on better describing the methods and evaluating the results more critically.*

**We discuss the implications of our results, but they remain secondary to our main goal of testing the prediction that most published results in medical journals are false.**

**Reviewer #2**:

*The manuscript entitled “Estimating the false discovery risk in medical journals based on published p-values” by Schimmack and Bartos, presents another attempt at estimating the risk of false discovery in the biomedical sciences. The authors do make some improvements over previous attempts, and I commend them for their rigor. However, I am not sure it really matters if the FDR is 50% or 20% or 12%. What matters is that we understand the factors contributing to a false discovery and train scientists to avoid them. The authors should address the following:*

**We were a bit surprised about the claim that the magnitude of the false positive rate is not important (does not matter). Taking this point of view to the extreme would mean we should abandon statistical significance testing, concerns about error rates, and just publish everything. While some people have advocated for such radical reforms, we believe that most scientists and consumers of scientific information want to have some assurance that they can trust published results to be more than just statistical noise. After all, Ioannidis’s article had such a big effect because it raised concerns that most published results are false. If that doesn’t really matter, why would his article have such a big effect?**

*1) The primary contributor to false discoveries in the biomedical sciences is low statistical power. The finding that the risk of false discovery is lower than predicted in a particular sample of a particular subset of the biomedical literature using scraping methods that, by the authors own admission, are prone to error is not really very informative. And it does not change that fact that many studies are underpowered and therefore are likely to indicate false discoveries. My concern is that this manuscript will be viewed as a vindication of current (flawed) research practices. Perhaps rewriting the introduction and discussion to take a more balanced approach to improving the quality of research rather than a somewhat combative approach to disproving previous estimates might be beneficial.*

Like Ioannidis’s article, Reviewer 2 argues from a position of assumptions. The claim is that we know that many studies are underpowered, but how do we know this? Our article shows that we can actually estimate the average power of studies and provide an estimate of the false positive risk. Rather than making assumptions, these results are based on empirical data. Surely, there can be issues with the data and we discuss these limitations, but estimates based on data are superior to claims based purely on assumptions. We find that the average power of clinical trials with significant results reported in the journals that we analyzed is NOT low, but somewhere between 60 and 80 percent, depending on assumptions about the percentage of false positives (more false positives = higher power for true findings).

**The main goal of our article is to replace guesses based on assumptions with empirical results. Whether 14% false positive results is high or low depends on scientists’ preferences for risk and we discuss how this percentage can be reduced by changing alpha. The main goal of our article is not to be combative or to make recommendation about research practices, but to provide the best possible estimate of the false positive risk for clinical trials published in leading medical journals, including Plos Medicine.**

*2) On page 7 the authors state that their estimate of the success of actual replication studies will be somewhere between 30 and 65%. Shouldn’t the false discovery risk be commensurate with these values since a failure to replicated would be considered to indicate a false discovery? It is not clear how these 2 parameters (replication success and initial probability of a false discovery) could be disconnected.*

**There are two explanations for a replication failure. One possibility is that the original study reported a false positive result. The other possibility is that the original study showed a true positive result, but the replication study produced a false negative result. The probability of the latter finding increases as power decreases. There are also other factors that can produce non-significant results in replication studies that are never exact including experimenter mistakes or moderating factors. In short, the percentage of false positive results is much smaller than the percentage of replication failures.**

*3) The suggestion on page 8 that the alpha value could be adjusted to reduce the risk of false positives is not very good advice. A more stringent alpha level applied to underpowered studies will lead to an inflation of effect size. Wouldn’t it be better to simply suggest researchers appropriately power their studies?*

**We do not propose that researchers change their a priori alpha level. Rather we suggest that readers of scientific journals can adjust their alpha levels to decide whether they find the evidence sufficient to rule out a false positive result. Our results merely sow that the false positive risk is 14% with alpha = .05 and 4% with alpha = .01. This is valuable information for readers to make their own choices.**

*4) The manuscript is mostly well written, however there are some sentences that are hard to parse and appear to be missing a word or two. For example:*

*Page 2 under Simulation Study: “…,we used estimated power individual studies based…”*

*Page 4, first full paragraph: “…since the current implementation only allows to deal with…”*

*Page 4, 3rd full paragraph: “…underestimation problem might to exclude p-values…”*

**Thank you, fixed. Thank you for your comments. We hope that our explanations and the revision clarify some of the misunderstandings of our initial manuscript.**

**Reviewer #3:**

*The authors aim to estimate the false discovery risk (the maximum false discovery rate) in medical literature, by analyzing p-values reported in PubMed abstracts. The authors approach seems sound, and I would like to applaud the fact that the authors have made all their code and study artifacts publicly available. However, I do have some concerns, which I hope the authors can address:*

*Major:*

*On page 5, the authors mention that they only extract p-values from abstracts labeled as “randomized clinical trial” or “clinical trial”. I think this is an important qualification that is currently easy to miss. Arguably, clinical trials are less likely to have publication bias and p-hacking, because they are expensive (and therefore less likely to be buried), often must be pre-registered, and typically require oversight by regulators. In fact, if I regenerate Figure 4 using data from my field (observational research), the cutoff at z = 1.96 is far more extreme than what the authors found for trials. I think this nuance is so important it should be mentioned in the title of the manuscript.*

Thank you for sharing the results of your own z-curve study. We hope that our article encourages further research of other research areas. We agree that our manuscript did not carefully distinguish between clinical trials and other studies and we revised it accordingly. Most important, we limit our discussion to clinical trials and compare our results to predictions made by Ioannidis for clinical trials. Our results suggest indeed that most clinical trials reported in the journals we analyzed have a high percentage of true hypothesis and good power. Whether other fields show lower power and higher false discovery risks is an important question to examine in future studies.

*I think the organization of the manuscript can be greatly improved. Currently, there is no distinction between introduction and methods, and the authors directly dive into simulations without explaining what their approach is that they’re evaluating in simulations. The authors currently assume the readers are completely knowledgeable of what was written in their z-curve paper, and the paper by Jagger and Leek. It would be good to summarize what was in these papers, and not explain what the authors did just by explaining how their approach differs from Jagger and Leek (since most readers, like me, will have forgotten the details of that paper).*

**We created a dedicated methods section where we describe both the False discovery risk, and the Z-curve method in greater detail. We now also explain that the Jagger and Leek’s model is a mixture of truncated beta distributions estimated on the *p*-values.**

Minor:

*Page 2, line 48: the term ‘z-curve’ needs to be introduced. I can guess it is the method referred to in the previous sentence, but that should be made explicit.*

**Fixed**

*Page 2, line 82: “we used estimated power individual studies based on medical meta-analysis.” I’m not sure what the authors mean by this. Also, I think this sentence is grammatically incorrect.*

**We edited the paragraph:**

**“we simulated H1 *p*-values from two-sided *z*-tests. We drew power of each simulated *z*-test (the only required parameter) from a distribution based on Lamberink and colleagues' (2018) empirical estimate of medical clinical trials' power (excluding all power estimates based on meta-analyses with non-significant results).”**

*Page 3, line 68: “We manipulated the true false discovery rate from 0 to 1…”. Please explain how.*

**We extended the sentence to explain that we did so by modifying the proportion of p-values simulated from the null and the alternative hypothesis.**

*Page 5, line 142.5: “the ‘Extractable’ column” Note that this column is called ‘Scrapeable’ in Table 2.*

**Thank you, fixed.**
