# Supplementary material for: Estimating the false discovery risk of (randomized) clinical trials in medical journals based on published p-values
Source: PLoS One. 2023 Aug 30;18(8):e0290084. doi: 10.1371/journal.pone.0290084 (PMC10468063; doi:10.1371/journal.pone.0290084)
Supplement: Attachment — Submitted filename: PLOS-response.docx [file pone.0290084.s002.docx]

Editor comment:

One of the reviewers has judged your manuscript to now be acceptable for the publication however, the other reviewer feels that their comments were not adequately addressed. Please could you review their comments and either incorporate the suggested revisions or provide clear justification for not doing so. You may even wish to discuss in your manuscript some of the critiques that the reviewer has flagged - they have raised some significant concerns about the messages that they are deriving from the manuscript.

**Thank you for the positive evaluation of the manuscript. Bellow, we address the remaining comments and incorporate them into the manuscript. In the end, we all shared the common goal of improving scientific practices and we carefully edited the take home messages that readers could derive from the manuscript.**

Reviewer comments to Author:

Reviewer #1:

(No Response)

Reviewer #2:

1) I was a bit surprised that the authors took a simple statement regarding the magnitude of the false discovery rate to not matter and turned into an abandonment of statistical significance testing. I must have not been as clear as necessary on making this point, so I will try to carefully reiterate and hopefully provoke a less defensive response.

I believe that we would agree that there is currently a lack of statistical training and rigor in many branches of the life sciences. Underpowered studies, which I and most researchers define as less than 80% power are common, leading to more false discoveries than expected. Let’s set aside the magnitude of this effect for the moment and focus on the authors statement made in their response that “consumers of scientific information want to have some assurance that they can trust published results to be more than just statistical noise.” Indeed, we agree on this. However, as a consumer of scientific information myself, I would feel (and am) much more comfortable reading a journal that requires the authors to describe statistical power and how it was derived than I would be reading a journal that just assumes all is well because the FDR is only 20%, not 60%.

The authors seem to be arguing with Ioannidis in their response. While his work may have been more theoretical, and therefore require certain assumptions, it cannot be denied that his intentionally provocative and seminal article has led to an increased interest in statistical rigor and has, as the authors state, led to the emergence of the field of meta-science in which they are currently working. Given this, I maintain that it is irresponsible to publish an analysis of false discovery risk predicted in a particular sample of a particular subset of the biomedical literature using scraping methods that, by the authors own admission, are prone to error without reinforcing the importance of conducting well powered studies. I strongly advise the authors to consider adding a few lines to their discussion simply making that clear.

**We quantified some of our claims to make clearer that our analysis holds for top medical journals:**

***“Thus, when considering top medical journals, a simple explanation for the discrepancy between Ioannidis's claim and our results is that Ioannidis's underestimated the proportion of well-powered studies in the literature.”***

***“Our article builds on these efforts by introducing an improved method to estimate the false discovery risk in top medical journals, with a focus on clinical trials.”***

***“Although our results provide some reassurance that medical research published in top journals is more robust than Ioannidis predicted, the false discovery risk in other medical journals and life sciences might be higher than our estimate.”***

**We also added the following sentence to clarify that the current practices are still not ideal and could be further improved.**

***“Importantly, our results do not warrant blind complacency when conducting and evaluating research or shifting focus from conducting well designed and highly powered studies.***”

2) The authors point on alpha of 0.05 vs 0.01 is confusing and needs to be better stated or removed. Despite their rebuttal statement that they “do not propose that researchers change their a priori alpha level”, they clearly seem to think that they should. They state that “To lower the FDR to 5% or less it is necessary to lower alpha” and imply a change in the a priori alpha value by stating “while journals may be reluctant to impose a lower level of alpha…” The authors fail to recognize the issue of effect size inflation and impact of this on future replication studies. The fact that “many researchers falsely assume that the alpha = .05 ensures a false discovery risk of only 5%” is again a problem in statistical training and rigor. Wouldn’t it be far better to suggest that readers actually understand what the alpha value means rather than suggest they calibrate to some personal alpha level? Choosing to ignore a result from a properly powered study with alpha =0.03 is bad advice and not good statistical practice.

**We edited the last paragraph to provide clearer explanation of alpha and its difference from false discovery risk.**

***“Many researchers falsely assume that the alpha = .05 ensures a false discovery risk of only 5%. However, the proportion of false positives equals alpha only when all tests are performed on true null hypotheses and all results are published. In the real world, the false positive rate depends on the proportion of true null hypotheses and the statistical power of studies examining true alternative hypotheses. We showed that in top medical journals alpha = .05 entails an FDR of 14%.”***
